# Eosinophil count testing in patients with asthma varies by healthcare provider type in the US: a retrospective study

**DOI:** 10.1186/s13223-024-00917-4

**Published:** 2024-10-24

**Authors:** Sameer Mathur, Thomas Corbridge, Elizabeth Packnett, Krutika Jariwala-Parikh, Arijita Deb

**Affiliations:** 1 University of Wisconsin School of Medicine and Public Health, Madison, WI USA; 2 Medical Affairs, GSK, Durham, NC USA; 3 Merative, Ann Arbor, MI USA; 4 US Value Evidence and Outcomes, GSK, Upper Providence, PA USA

**Keywords:** Asthma, Biologic, Eosinophil testing, Exacerbations, Healthcare provider

## Abstract

**Background:**

Patients with asthma with an eosinophilic phenotype may be eligible for additional treatment options to improve disease control; however, the prevalence and frequency of eosinophil testing is unknown. This study assessed blood eosinophil count testing prevalence in patients with asthma by exacerbation frequency and healthcare provider (HCP) type.

**Methods:**

This was a retrospective, longitudinal, real-world study (GSK ID: 214470) utilizing the Merative Explorys^®^ Universe electronic health records database. Eligible patients had ≥ 2 asthma diagnostic codes (January 2016–December 2018) (Index date: first asthma diagnosis). Outcomes included patient demographics and clinical characteristics (12 months pre-index [baseline]), and prevalence of blood eosinophil count testing, stratified by exacerbation frequency (infrequent exacerbations [< 2]) or frequent exacerbations [≥ 2] or primary HCP (Allergist/Pulmonologist, a primary care physician [PCP] or other HCP) during the 12 months post-index (follow-up).

**Results:**

Of 400,254 patients included (mean age: 51.2 years; 70.8% female), the most common provider type at baseline was a PCP (76.8%). A higher proportion of patients with frequent exacerbations had blood eosinophil count tests at baseline (55.4–69.5%) and follow-up (67.9–75.1%), compared with patients with infrequent exacerbations (55.5–63.7%, 62.4–67.3%). Significantly more patients in the Allergist/Pulmonologist subgroup had ≥ 1 blood eosinophil count test result compared with patients in the PCP subgroup at both baseline (59.9% vs. 50.7%; *p* < 0.001) and follow-up (59.0% vs. 56.2%; *p* < 0.001). In the total population, the mean (SD) number of tests ordered was 3.4 (5.3) and 4.1 (6.4) during the baseline and follow-up periods, respectively. A greater mean number of tests were ordered for patients with frequent exacerbations, most apparently in the Allergist/Pulmonologist subgroup during baseline and follow-up (7.4 vs. 4.9). For patients with frequent exacerbations and blood eosinophil count test results, the mean (SD) number of tests ranged from 3.1 (4.6) to 5.8 (8.3) at baseline and 5.1 (8.5) to 7.4 (10.6) during follow-up.

**Conclusions:**

The prevalence of blood eosinophil count testing in patients with asthma remains suboptimal. Routine blood eosinophil count testing should be considered by HCPs for patients with asthma to increase identification of the eosinophilic asthma phenotype, which may inform the decision to advance to targeted biologic therapy.

**Supplementary Information:**

The online version contains supplementary material available at 10.1186/s13223-024-00917-4.

## Introduction

Multiple phenotypes of asthma have now been identified, including one characterized by eosinophilic inflammation [1]. Despite treatment with standard of care therapies, patients with asthma with an eosinophilic phenotype report poor lung function and are at greater risk of exacerbations compared with patients with non-eosinophilic asthma [2, 3]. Although previous prevalence estimates suggest that 50% of patients with severe asthma have an eosinophilic phenotype [1, 4], this may be an underestimate; a recent study by Heaney et al. indicated that as many as 84% of patients internationally with severe asthma may have an eosinophilic phenotype [4].

Identifying an eosinophilic phenotype in patients with moderate-to-severe asthma enhances therapeutic options, allowing for targeted therapy eligibility to be established [1]. Targeted therapies reduce clinically significant exacerbation frequency and oral corticosteroid (OCS) dependence [5–14], in addition to improvements in asthma control, lung function, and health-related quality of life (HRQoL) versus placebo [6–12, 14]. Consequently, current Global Initiative for Asthma (GINA) 2022 recommendations suggest testing blood eosinophil counts to facilitate asthma phenotype identification [1]. Given the variability in blood eosinophil counts between readings, specific recommendations include repeating blood eosinophil count tests up to three times independently, at least 1–2 weeks after OCS use or while on the lowest possible OCS dose, as counts ≥ 150 cells/µL suggest Type 2 (T2) airway inflammation [1]. Although blood eosinophil count measurement is performed as part of the differential component of a complete blood count (CBC), data on the prevalence of testing for patients with asthma in the real-world setting are limited. Of note, the guidelines also recommend referral to specialist care for patients with severe asthma at any stage of treatment [1]. It is unknown whether blood eosinophil testing prevalence differs by healthcare provider (HCP) type and patient exacerbation frequency.

The objective of this study was to assess the real-world prevalence and frequency of blood eosinophil count testing in patients with asthma by patient exacerbation frequency and HCP type.

## Materials and methods

### Study design

**Fig. 1:**
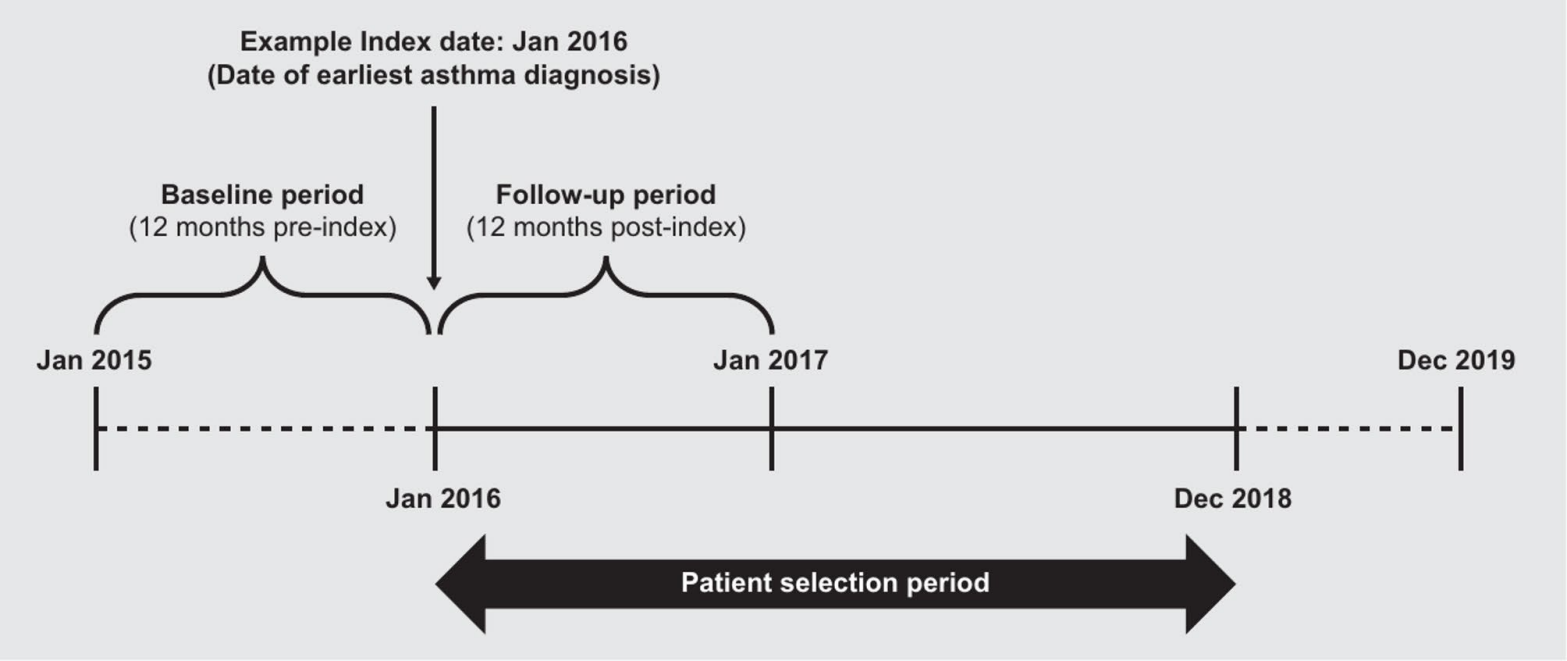
Study design

This was a retrospective, longitudinal, real-world study (GSK ID: 214470) utilizing the Merative Explorys^®^Universe electronic health records database. The Merative Explorys^®^ Universe includes electronic health records, billing, and claims data from public and commercial payers, representing approximately 18% of the US population. The study used data from January 01, 2015 to December 31, 2019 (Fig. 1). The index date was the date of first asthma diagnosis code between January 01, 2016 and December 31, 2018. The baseline and follow-up periods were defined as fixed 12 months pre- and post-index date, respectively.

The data records were de-identified and fully compliant with US patient confidentiality requirements, including the Health Insurance Portability and Accountability Act (HIPAA) of 1996. This study used only de-identified patient records and did not involve the collection, use or transmittal of individually identifiable data; thus, Institutional Review Board (IRB) approval was not necessary.

All outcomes/variables were assessed in the total population, by HCP type (Allergist/Pulmonologist [16.5%], primary care physician (PCP) [76.8%], or other HCP (defined as any provider type that was not Allergist, Pulmonologist, PCP, or missing) [6.7%]) during the baseline period and exacerbation frequency during the follow-up period (exacerbations definition described in **Outcomes and variables**). If patients saw more than one HCP during the baseline period, patients were hierarchically assigned, with preference given to Allergist/Pulmonologist, followed by PCP, and then other HCP. Patients with missing provider type on any outpatient encounters during the baseline period were excluded. Blood eosinophil count outcomes in patients with ≥ 2 exacerbations (frequent) or < 2 exacerbations (infrequent) in baseline and follow-up periods were also further assessed in subgroups of patients by HCP type.

### Patient population

Eligible patients were ≥ 12 years of age at index, had at least two diagnostic codes (International classification of diseases [ICD]-9, ICD-10 or Systematized Nomenclature of Medicine [SNOMED] clinical terms (**Supplementary Table 1**) for asthma at least one day apart between January 1, 2016, and December 31, 2018, had at least one outpatient visit during both the baseline and the follow-up periods and had laboratory data available as identified by their respective logical observations identifiers names and codes (LOINC), current procedural terminology codes (CPT), and SNOMED Clinical Terms (**Supplementary Table 2**).

### Outcomes and variables

Patient demographics and baseline characteristics assessed included age, sex, race, insurance type, US geographic region, asthma exacerbations, Deyo-Charlson Comorbidity Index (DCI), comorbid conditions, asthma biologic therapy use and other asthma treatment use. For this study, 16 comorbid conditions were also assessed and identified by the presence of a diagnosis code during the baseline period.

Exacerbations were identified by the presence of a medical claim with a diagnostic code for asthma and at least one prescription claim for systemic corticosteroids (intramuscular, intravenous, or oral; detailed within **Supplementary Table 3**) within the 5 days before or after the asthma diagnostic code. Multiple exacerbations meeting this definition within 14 days were considered a single exacerbation event [15].

Assessments of the prevalence of blood eosinophil count testing included the proportion of patients with at least one blood eosinophil count or CBC test with differential, and for patients with at least one test result, the maximum blood eosinophil count (< 150, 150–299, or ≥ 300 cells/µL) during both the baseline and follow-up periods. Prescriptions for targeted biologic therapies (benralizumab, dupilumab, mepolizumab, omalizumab, and reslizumab) and other prescribed asthma treatments (inhaled corticosteroids [ICS], OCS, short-acting β_2_-agonists [SABA], short-acting muscarinic antagonists [SAMA], long-acting β_2_-agonists [LABA], long-acting muscarinic antagonists [LAMA], leukotriene receptor antagonists [LTRA], ICS/LABA, triple therapy [ICS + LABA + LAMA]) were also assessed during the baseline and follow-up periods.

### Sample size and statistical analyses

All outcomes were assessed descriptively, with continuous variables presented as means with standard deviations (SD) and categorical variables were presented as counts and percentages. Chi-squared tests and t-tests were used to test for differences (Allergist/Pulmonologist versus PCP; Allergist/Pulmonologist versus other HCP) in nominal/categorical variables and interval/continuous variables, respectively, with a p-value < 0.05 considered statistically significant. Statistical testing was performed using World Programming System 4.02.

## Results

### Patient population

Of the 856,779 patients with at least two diagnostic codes for asthma during the study period, 400,254 (46.7%) were eligible for inclusion in the total study population (**Supplementary Fig. 1**). When split by HCP type, 16.5% (*n* = 66,040) of patients were under the care of an Allergist/Pulmonologist, 76.8% (*n* = 307,308) a PCP, and 6.7% (*n* = 26,906) by other types of HCP.

### Patient demographics and baseline characteristics

The mean (SD) age of the total study population was 51.2 (19.5), the majority were female (70.8%), Caucasian (74.5%), and from the North Central region of the US (59.3%) (Table 1). These data were generally similar across exacerbation subgroups, although the proportion of African American patients was slightly higher in the patients with frequent exacerbations (20.3%) versus infrequent exacerbations (18.0%) (Table 1). When analyzed by HCP type, patients under Allergist/Pulmonologist versus PCP or other HCP care had a significantly higher mean (SD) age (58.2 [17.9] vs. 50.2 [19.5] and 45.7 [18.9]; *p* < 0.001 for each comparison), and a significantly lower proportion were female (68.7% vs. 71.1% and 73.4%; *p* < 0.001 for each comparison). A significantly higher proportion of patients under Allergist/Pulmonologist versus PCP or other HCP care were Caucasian (76.8% vs. 74.6% and 68.4%, *p* < 0.001) and had Medicare insurance (30.9% vs. 20.9% and 17.9%, *p* < 0.001) (Table 2). A higher proportion of Caucasian (17.0%) and Asian (18.1%) patients were under Allergist/Pulmonologist care than African American (15.3%) and Hispanic/Latino patients (13.6%), whereas a higher proportion of African American (8.9%) and Hispanic/Latino (8.4%) patients had other HCP care when compared with Caucasian (6.2%) and Asian (5.0%) patients.

**Table 1:** Patient baseline demographics and clinical characteristics by exacerbation frequency

	Total population (*N* = 400,254)	Patients with infrequent (< 2) exacerbations* (*n* = 374,657)	Patients with frequent (≥ 2) exacerbations* (*n* = 25,597)
**Age**,** years**,** mean (SD)**	51.2 (19.5)	51.2 (19.6)	51.2 (18.6)
**Female**,** n (%)**	283,525 (70.8)	265,263 (70.8)	18,262 (71.3)
**Race**,** n (%)**			
Hispanic/Latino	2943 (0.7)	2677 (0.7)	266 (1.0)
African American	72,602 (18.1)	67,397 (18.0)	5,205 (20.3)
Caucasian	298,308 (74.5)	279,746 (74.7)	18,562 (72.5)
Asian	3631 (0.9)	3419 (0.9)	212 (0.8)
Other/Unknown/Refused	22,770 (5.7)	21,418 (5.7)	1352 (5.3)
**Insurance type**,** n (%)**			
Private	175,797 (43.9)	164,678 (44.0)	11,119 (43.4)
Medicare	89,516 (22.4)	84,033 (22.4)	5483 (21.4)
Medicaid	49,105 (12.3)	46,087 (12.3)	3018 (11.8)
Other/Self-pay/Unknown	85,836 (21.5)	79,859 (21.3)	5977 (23.4)
**Geographic region n (%)**			
Northeast	13,337 (3.3)	12,649 (3.4)	688 (2.7)
North Central	237,498 (59.3)	221,360 (59.1)	16,138 (63.1)
South	109,736 (27.4)	102,687 (27.4)	7049 (27.5)
West	39,018 (9.8)	37,314 (10.0)	1704 (6.7)
Unknown	665 (0.2)	647 (0.2)	18 (0.1)
**DCI score**,** mean (SD)**	1.3 (1.8)	1.3 (1.8)	1.4 (1.7)

No statistical analysis was performed to compare between patients stratified by exacerbation frequency; *Within the follow-up period (12 month post-index), as identified by the presence of an asthma diagnosis and at least one prescription for systemic corticosteroids (IM, IV, or oral) 5 days pre or post diagnosis; exacerbations occurring within 14 days were considered a single exacerbation. All demographics were captured at index date

DCI, Deyo-Charlson Comorbidity Index; IM, intramuscular; IV, intravenous; SD, standard deviation

**Table 2:** Patient demographics and baseline characteristics by HCP type

	Total population (*N* = 400,254)	Allergist/Pulmonologist (*n* = 66,040)	PCP (*n* = 307,308)	Other HCP (*n* = 26,906)
**Age**,** years**,** mean (SD)**	51.2 (19.5)	58.2 (17.9)	50.2 (19.5)***	45.7 (18.9)***
**Female**,** n (%)**	283,525 (70.8)	45,373 (68.7)	218,395 (71.1)***	19,757 (73.4)***
**Race**,** n (%)**			*** ^**†**^	*** ^**†**^
Hispanic/Latino	2943 (0.7)	400 (0.6)	2297 (0.8)	246 (0.9)
African American	72,602 (18.1)	11,140 (16.9)	55,032 (17.9)	6430 (23.9)
Caucasian	298,308 (74.5)	50,703 (76.8)	229,216 (74.6)	18,389 (68.4)
Asian	3631 (0.9)	659 (1.0)	2790 (0.9)	182 (0.7)
Other/Unknown/Refused	22,770 (5.7)	3138 (4.8)	17,973 (5.9)	1659 (6.2)
**Insurance type**,** n (%)**			***^†^	***^†^
Private	175,797 (43.9)	26,956 (40.8)	138,982 (45.2)	9859 (36.6)
Medicare	89,516 (22.4)	20,377 (30.9)	64,332 (20.9)	4807 (17.9)
Medicaid	49,105 (12.3)	5495 (8.3)	38,081 (12.4)	5529 (20.6)
Other/Self-pay/Unknown	85,836 (21.5)	13,212 (20.0)	65,913 (21.5)	6711 (24.9)
**Geographic region**,** n (%)**			***^†^	***^†^
Northeast	13,337 (3.3)	2949 (4.5)	8350 (2.7)	2038 (7.6)
North Central	237,498 (59.3)	39,239 (59.4)	184,523 (60.0)	13,736 (51.1)
South	109,736 (27.4)	17,405 (26.4)	85,626 (27.9)	6705 (24.9)
West	39,018 (9.8)	6334 (9.6)	28,394 (9.2)	4290 (15.9)
Unknown	665 (0.2)	113 (0.2)	415 (0.1)	137 (0.5)
**Asthma exacerbations in the year prior to index**,** n (%)**	34,810 (8.7)	11,185 (16.9)	22,497 (7.3)***	1128 (4.2)***
Mean (SD)	1.4 (0.9)	1.5 (1.0)	1.3 (0.8)***	1.4 (0.9)***
**DCI score**,** mean (SD)**	1.3 (1.8)	2.0 (2.1)	1.2 (1.7)***	0.8 (1.5)***

All demographics measured at index date. Other HCP category includes any provider type that was not Allergist, Pulmonologist, PCP, or missing

***p < 0.001 vs. Allergist/Pulmonologist subgroup; ^**†**^positive p-value indicates that the distribution by race/insurance type/geographic region was significantly different vs. Allergist/Pulmonologist subgroup

DCI, Deyo-Charlson Comorbidity Index; HCP, healthcare provider; PCP, primary care physician; SD, standard deviation

When analyzed by HCP, patients under Allergist/Pulmonologist care had the highest mean (SD) number of exacerbations (1.5 [1.0]) compared with 1.3 (0.8) and 1.4 (0.9) for those under the care of PCPs and other HCP, respectively (*p* < 0.001 for each comparison) (Table 2). The mean (SD) DCI score was 1.3 (1.8) in the total population and was similar across exacerbation subgroups (Table 1). When analyzed by HCP type, patients in the Allergist/Pulmonologist subgroup had a significantly higher comorbidity score (DCI score: 2.0 [2.1]) compared with PCP or other HCP subgroups (DCI score: 1.2 [1.7] and 0.8 [1.5], respectively; *p* < 0.001 for each comparison) (Table 2).

**Fig. 2:**
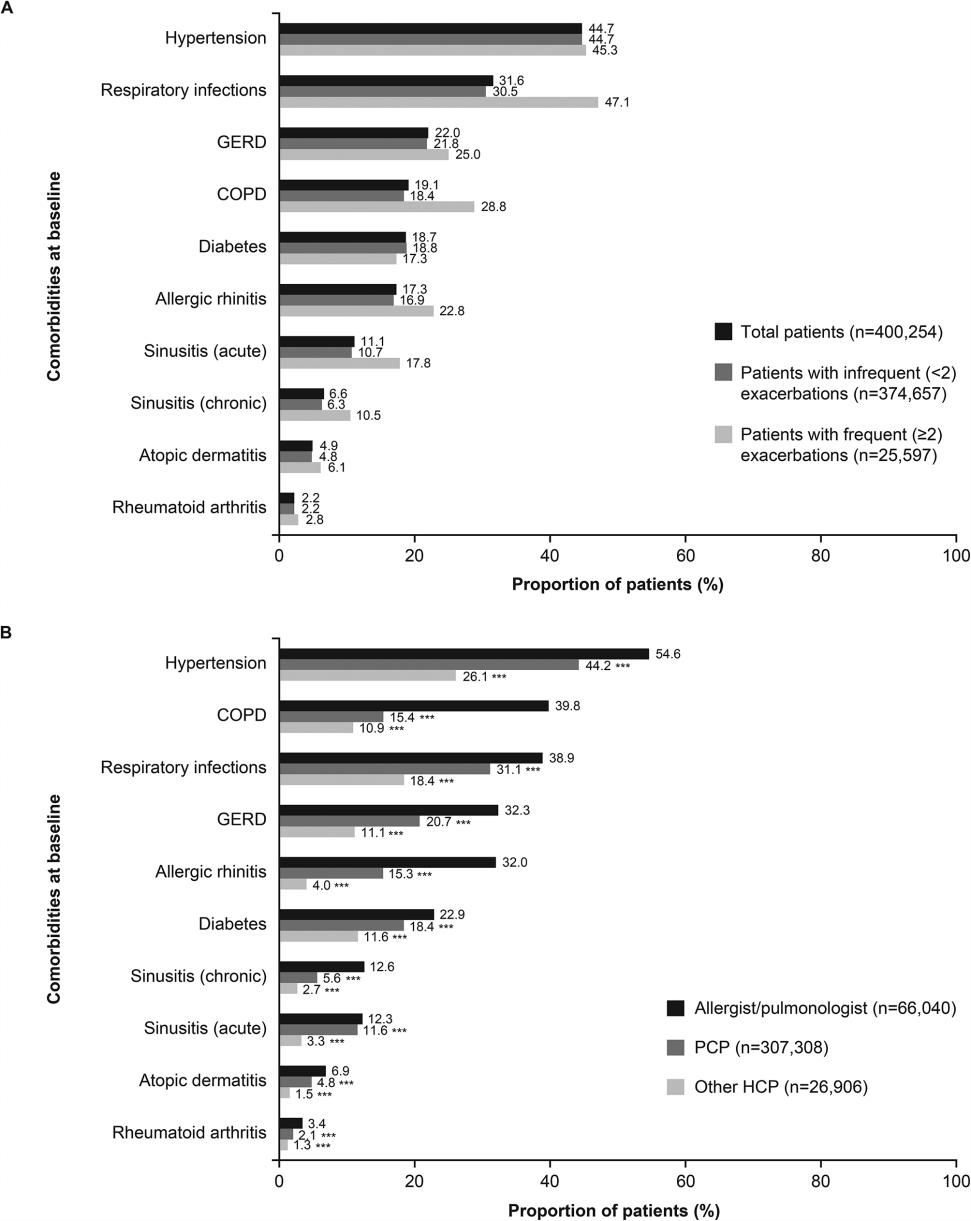
Proportion of patients with the top ten specified comorbidities^**†**^ during the baseline period by exacerbation frequency (**A**) and HCP type (**B**) No statistical analysis was performed to compare between patients stratified by exacerbation frequency; ***P < 0.001 vs. Allergist/Pulmonologist subgroup; ^†^Proportion of total patients with each of the other six pre-specified comorbidities was < 1% (not presented here) COPD, chronic obstructive pulmonary disease; GERD, gastroesophageal reflux disease; HCP, healthcare provider; PCP, primary care physician

During the baseline period, the most common comorbidities in the total population were hypertension (44.7%), respiratory infections (31.6%), and gastroesophageal reflux disease (GERD) (22.0%) (Fig. 2A). For patients with frequent exacerbations during the follow-up period, the most common comorbidities were respiratory infections (47.1%), hypertension (45.3%), and chronic obstructive pulmonary disease (COPD) (28.8%). Similar to the total population, for patients with infrequent exacerbations during the follow-up period, the most common comorbidities were also hypertension (44.7%), respiratory infections (30.5%), and GERD (21.8%) (Fig. 2A). The most common comorbidities at baseline for patients per HCP subgroup included hypertension (54.6%), COPD (39.8%), and respiratory infections (38.9%) in the Allergist/Pulmonologist subgroup, hypertension (44.2%), respiratory infections (31.1%), and GERD (20.7%) in the PCP subgroup and hypertension (26.1%), respiratory infections (18.4%), and diabetes (11.6%) in the other HCP subgroup (Fig. 2B). A significantly higher proportion of patients in the Allergist/Pulmonologist subgroup than the PCP or other HCP subgroup (*p* < 0.001) reported comorbidities, across all comorbidities assessed.

### Exacerbations during the follow-up period

**Fig. 3:**
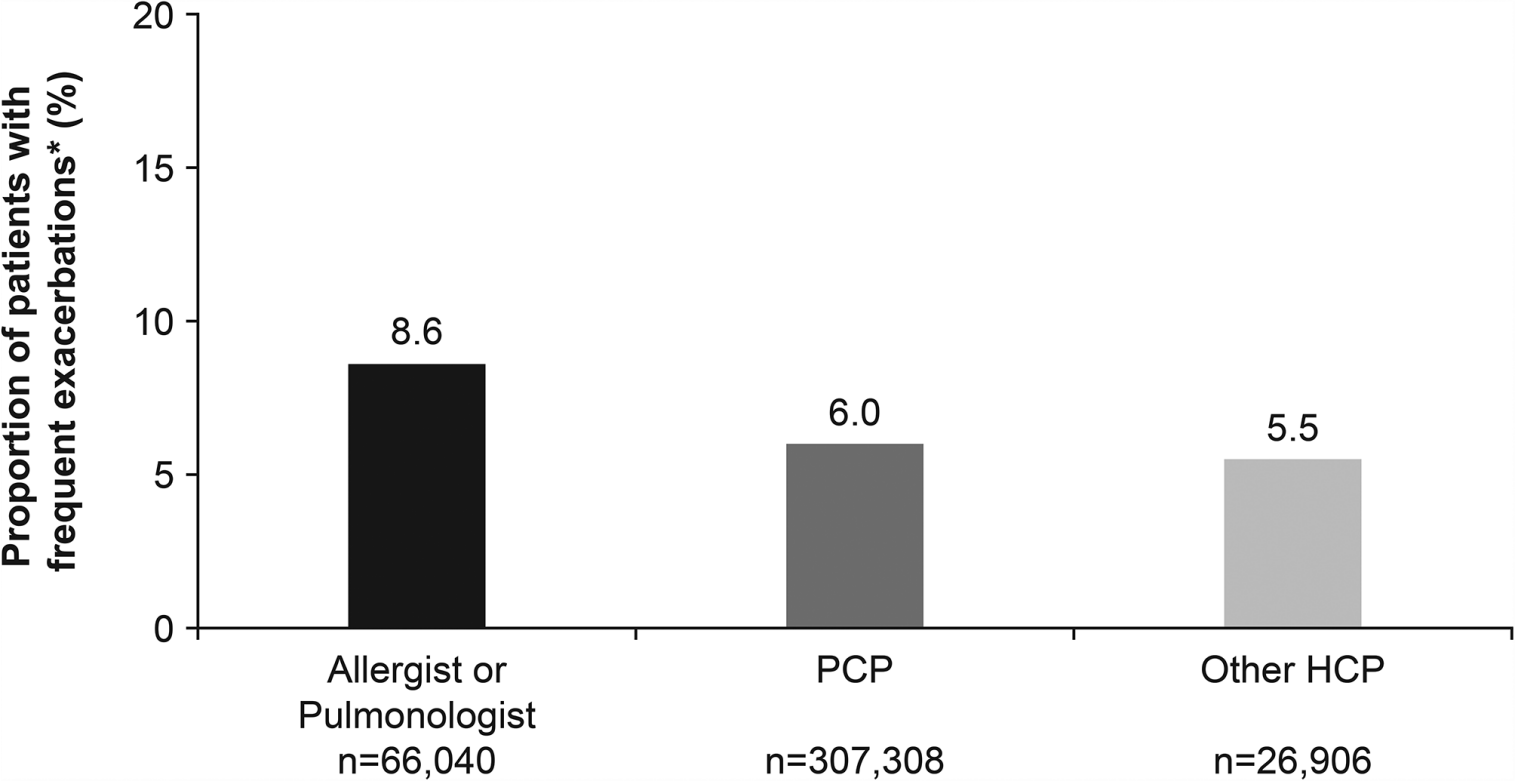
Proportion of patients with frequent (≥ 2) exacerbations during the follow-up period by HCP type No statistical analysis was performed; *Of total patients within the relevant HCP subgroup HCP, healthcare provider; PCP, primary care physician

During the follow-up period, 6.4% of patients in the total population experienced frequent asthma exacerbations. The proportion of patients with frequent exacerbations during the follow-up period was 8.6% in the Allergist/Pulmonologist subgroup, 6.0% in the PCP subgroup and 5.5% in the other HCP subgroup (Fig. 3).

### Blood eosinophil count testing and results

**Fig. 4:**
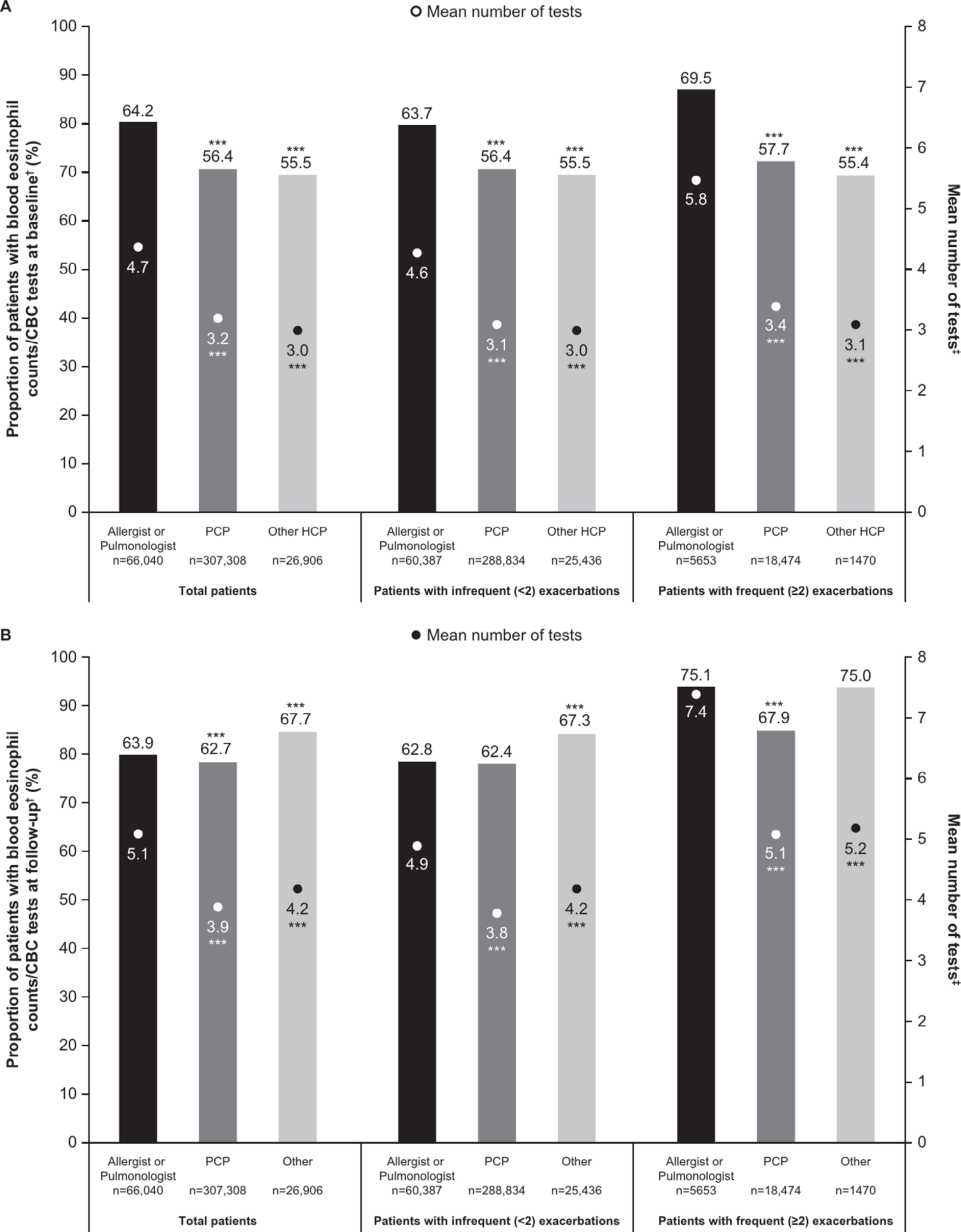
Proportion of patients with ≥ 1 blood eosinophil count/CBC test and the mean number of tests ordered at baseline (**A**) and follow-up (**B**) No statistical analysis was performed to compare between patients stratified by exacerbation frequency; ***p < 0.001 vs. Allergist/Pulmonologist subgroup; ^†^Presence of blood eosinophil counts or CBC testing ordered; ^‡^Number of distinct days with eosinophil or CBC tests ordered among patients with at least one test ordered CBC, complete blood count; HCP, healthcare provider; PCP, primary care physician

In the total population, 57.7% (*n* = 230,773) and 63.2% (*n* = 253,137) of patients had at least one blood eosinophil count/CBC test during the baseline and follow-up periods, respectively. During the baseline period, a significantly higher proportion of patients in the Allergist/Pulmonologist subgroup had a blood eosinophil count/CBC count (64.2%) compared with the PCP (56.4%) and other HCP (55.5%) subgroups (*p* < 0.001 for each comparison) (Fig. 4A). During the follow-up period, the proportion of patients in the Allergist/Pulmonologist (63.9%) subgroup with a blood eosinophil count/CBC count test was significantly higher compared with patients in the PCP (62.7%) subgroup and significantly lower compared with the other HCP subgroup (67.7%) (*p* < 0.001 for each comparison) (Fig. 4B**).**

When analyzed by exacerbation frequency during the follow-up period, in patients with frequent exacerbations, 30.5%, 42.3%, and 44.6% did not have a blood eosinophil count or CBC ordered in the Allergist/Pulmonologist, PCP or other HCP subgroups, respectively, in the baseline period. Additionally, 24.9%, 32.1%, and 25.0% of patients with frequent exacerbations in the Allergist/Pulmonologist, PCP or other HCP subgroups, respectively, did not have a test ordered in the follow-up period (Fig. 4). Overall, more patients with frequent exacerbations versus infrequent exacerbations had a blood eosinophil count/CBC test across all HCP types, with the exception of other HCPs during the baseline period; this trend was most apparent for the Allergist/Pulmonologist subgroup during the baseline (69.5% vs. 63.7%) and follow-up (75.1% vs. 62.8%) periods (Fig. 4).

In the total population, the mean (SD) number of tests ordered was 3.4 (5.3) and 4.1 (6.4) during the baseline and follow-up periods, respectively. Trends by HCP were similar to results for proportion of patients with a test, with mean use significantly higher for those patients in the Allergist/Pulmonologist subgroup compared with the PCP and other HCP subgroups, regardless of exacerbation subgroup (*p* < 0.001 for each comparison). The number of tests across exacerbation subgroups for patients under the care of an Allergist/Pulmonologist ranged from 4.6 to 5.8 tests during the baseline period and from 4.9 to 7.4 during the follow-up period (Fig. 4). When analyzed by exacerbation frequency, more tests were ordered for patients with frequent exacerbations versus infrequent exacerbations, across all provider subgroups during baseline and follow-up periods; this trend was most apparent for the Allergist/Pulmonologist subgroup during the baseline (5.8 vs. 4.6) and follow-up (7.4 vs. 4.9) periods (Fig. 4). For patients with frequent exacerbations who had at least one blood eosinophil count test ordered, the mean (SD) number of tests ranged from 3.1 (4.6) to 5.8 (8.3) at baseline and 5.1 (8.5) to 7.4 (10.6) during follow-up.

**Fig. 5:**
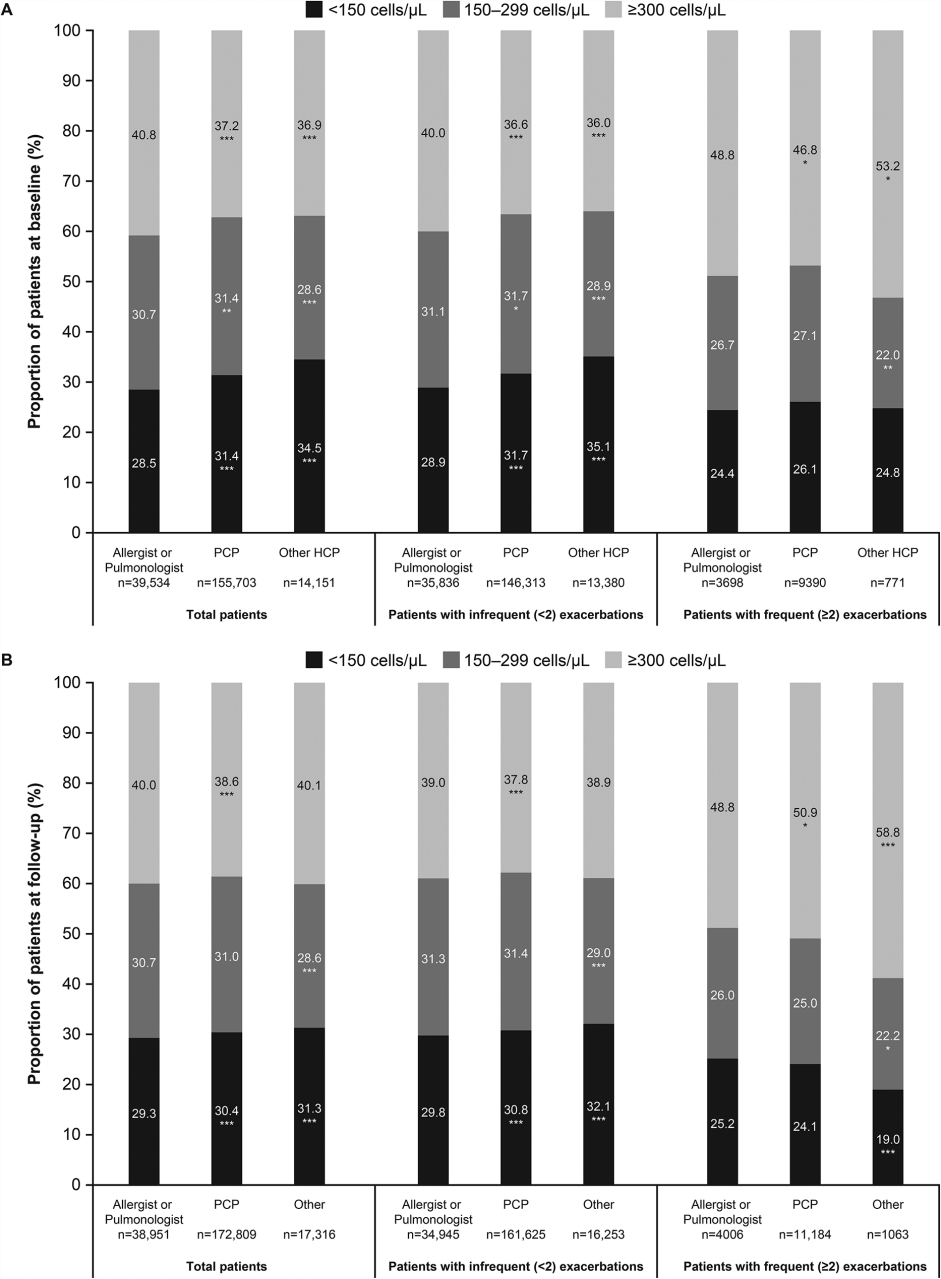
Maximum blood eosinophil count test results during the baseline (**A**) and follow-up (**B**) periods No statistical analysis was performed to compare between patients stratified by exacerbation frequency; *p < 0.05, **p < 0.01, and ***p < 0.001 vs. Allergist/Pulmonologist subgroup CBC, complete blood count; PCP, primary care physician

When comparing maximum blood eosinophil count results in the total population during the baseline period, 31.0% of patients had a maximum count of < 150 cells/µL, 31.1% 150–299 cells/µL and 37.9% ≥300 cells/µL. During the baseline period, for total patients, the Allergist/Pulmonologist subgroup had a significantly higher proportion of patients with maximum blood eosinophil counts ≥ 300 cells/µL (40.8%) compared with PCP (37.2%) and other HCP (36.9%) subgroups (*p* < 0.001 for each comparison) (Fig. 5A). When analyzed by exacerbation frequency, more patients with frequent exacerbations had maximum blood eosinophil counts ≥ 300 cells/µL (47.7%) compared with patients with infrequent exacerbations (37.2%). For patients with frequent exacerbations in the Allergist/Pulmonologist subgroup, a significantly higher proportion had maximum blood eosinophil counts ≥ 300 cells/µL (48.8%) compared with the PCP subgroup (46.8%; *p* < 0.05), but a significantly lower proportion compared with the other HCP subgroup (53.2%; *p* < 0.05) (Fig. 5A). For patients with infrequent exacerbations, the maximum blood eosinophil counts ≥ 300 cells/µL were significantly higher in the Allergist/Pulmonologist subgroup (40.0%) than the PCP (36.6%) and other HCP (36.0%) subgroups (*p* < 0.001 for each comparison) (Fig. 5A).

During the follow-up period, 30.2% of patients in the total population had a maximum count of < 150 cells/µL, 30.8% 150–299 cells/µL and 39.0% ≥ 300 cells/µL. When analyzed by HCP type, the Allergist/Pulmonologist subgroups had a significantly higher proportion of patients with blood eosinophil counts ≥ 300 cells/µL (40.0%) compared with the PCP (38.6%) subgroup but a similar proportion to the other HCP subgroup (40.1%) (Fig. 5B). When analyzed by exacerbation frequency, more patients in the frequent exacerbation subgroup had blood eosinophil counts ≥ 300 cells/µL (50.9%) compared with the infrequent exacerbation subgroup (38.0%). For patients with frequent exacerbations in the Allergist/Pulmonologist subgroup, the proportion with blood eosinophil counts ≥ 300 cells/µL (48.8%) was significantly lower compared with the PCP (50.9%; *p* < 0.05) and other HCP subgroups (58.8%; *p* < 0.001) (Fig. 5B). For patients with infrequent exacerbations, a higher proportion of patients in the Allergist/Pulmonologist subgroup had maximum blood eosinophil counts ≥ 300 cells/µL (39.0%), compared with other HCP (38.9%; *p* = 0.869) and PCP (37.8%; *p* < 0.001) subgroups (Fig. 5B).

### Asthma medication

For the total population and for all HCP subgroups the proportion of patients receiving targeted biologic therapy increased from baseline to follow-up periods, the total population increased from 0.4 to 0.6%, Allergist/Pulmonologist increased from 1.7 to 2.1%, PCP increased from 0.1 to 0.2%, and other HCP increased from 0.2 to 0.5% (Table 3). The most frequently used biologic therapy across HCP subgroups was omalizumab during both the baseline and follow-up periods (Allergist/Pulmonologist 1.6–1.8%; PCP 0.1–0.2%; other providers 0.2–0.4%; Table 3). During the follow-up period, the use of asthma medications increased for all HCP subgroups compared with those at baseline, with the exception of SAMA and methylxanthines for patients within the Allergist/Pulmonologist subgroup (Table 3). Significantly more patients in the Allergist/Pulmonologist subgroup were receiving other asthma medications during the baseline or follow-up periods compared with those within the PCP or other HCP subgroups (non-significant for SABA + SAMA use in the follow-up period for the other HCP subgroup) (Table 3). Of the other asthma medications assessed, SABA was the most commonly used medication by patients during both the baseline and follow-up periods across provider subtype.

**Table 3:** Asthma medication by provider type

	Total population (*N* = 400,254)		Allergist/Pulmonologist (*n* = 66,040)		PCP (*n* = 307,308)		Other (*n* = 26,906)	
	Baseline period	Follow-up period	Baseline period	Follow-up period	Baseline period	Follow-up period	Baseline period	Follow-up period
**Asthma-related biologics**,** n (%)**	1507 (0.4)	2252 (0.6)	1100 (1.7)	1391 (2.1)	349 (0.1)***	737 (0.2)***	58 (0.2)***	124 (0.5)***
Benralizumab	1 (0.0)	40 (0.0)	1 (0.0)	16 (0.0)	0 (0.0)	22 (0.0)***	0 (0.0)	2 (0.0)
Dupilumab	4 (0.0)	42 (0.0)	1 (0.0)	10 (0.0)	3 (0.0)	32 (0.0)	0 (0.0)	0 (0.0)
Mepolizumab	29 (0.0)	362 (0.1)	19 (0.0)	210 (0.3)	10 (0.0)***	136 (0.0)***	0 (0.0)**	16 (0.1)***
Omalizumab	1473 (0.4)	1847 (0.5)	1079 (1.6)	1182 (1.8)	336 (0.1)***	559 (0.2)***	58 (0.2)***	106 (0.4)***
Reslizumab	1 (0.0)	12 (0.0)	1 (0.0)	6 (0.0)	0 (0.0)	4 (0.0)**	0 (0.0)	2 (0.0)
**Other asthma medications**,** n (%)**^**†**^								
ICS	32,150 (8.0)	47,092 (11.8)	9626 (14.6)	10,853 (16.4)	21,374 (7.0)***	34,109 (11.1)***	1150 (4.3)***	2130 (7.9)***
OCS	109,683 (27.4)	149,162 (37.3)	27,716 (42.0)	29,146 (44.1)	77,787 (25.3)***	112,188 (36.5)***	4180 (15.5)***	7828 (29.1)***
SABA	177,229 (44.3)	234,642 (58.6)	38,544 (58.4)	39,991 (60.6)	130,403 (42.4)***	181,229 (59.0)***	8282 (30.8)***	13,422 (49.9)***
SAMA	12,435 (3.1)	16,849 (4.2)	4621 (7.0)	4496 (6.8)	7228 (2.4)***	11,286 (3.7)***	586 (2.2)***	1067 (4.0)***
SABA + SAMA	74,348 (18.6)	103,691 (25.9)	18,161 (27.5)	19,123 (29.0)	51,849 (16.9)***	76,835 (25.0)***	4338 (16.1)***	7733 (28.7)
LABA	2009 (0.5)	2690 (0.7)	799 (1.2)	910 (1.4)	1105 (0.4)***	1606 (0.5)***	105 (0.4)***	174 (0.6)***
LAMA	8585 (2.1)	10,192 (2.5)	3966 (6.0)	4042 (6.1)	4285 (1.4)***	5586 (1.8)***	334 (1.2)***	564 (2.1)***
LTRA	58,342 (14.6)	80,737 (20.2)	16,210 (24.5)	18,078 (27.4)	40,295 (13.1)***	59,286 (19.3)***	1837 (6.8)***	3373 (12.5)***
ICS/LABA	60,130 (15.0)	85,235 (21.3)	19,039 (28.8)	21,678 (32.8)	39,018 (12.7)***	59,841 (19.5)***	2073 (7.7)***	3716 (13.8)***
Triple therapy (ICS + LABA + LAMA)	39,483 (9.9)	55,601 (13.9)	12,866 (19.5)	14,129 (21.4)	25,173 (8.2)***	38,854 (12.6)***	1444 (5.4)***	2618 (9.7)***
Mast cell stabilizers	147 (0.0)	194 (0.0)	82 (0.1)	89 (0.1)	63 (0.0)***	99 (0.0)***	2 (0.0)***	6 (0.0)***
Methylxanthines	2318 (0.6)	2375 (0.6)	975 (1.5)	932 (1.4)	1288 (0.4)***	1369 (0.4)***	55 (0.2)***	74 (0.3)***
LABA/LAMA	694 (0.2)	1451 (0.4)	314 (0.5)	545 (0.8)	353 (0.1)***	852 (0.3)***	27 (0.1)***	54 (0.2)***

**p < 0.01, and ***p < 0.001 vs. Allergist/Pulmonologist subgroup; ^†^Categories non-mutually exclusive;

ICS, inhaled corticosteroid; LABA, long-acting β2-agonist; LAMA, long-acting muscarinic antagonist; LTRA, leukotriene receptor antagonist; OCS, oral corticosteroids; SABA, short-acting β2-agonist; SAMA, short-acting muscarinic antagonist

When patients were categorized by exacerbation frequency during the follow-up period, the proportion of patients receiving targeted biologic therapies increased during baseline to follow-up from 1.2 to 2.2% in patients with frequent exacerbations and from 0.3 to 0.5% for patients with infrequent exacerbations (**Supplementary Table 4**). Omalizumab was the most frequently used biologic therapy during the baseline and follow-up period for patients with frequent exacerbations (1.2% and 1.7%) and patients with infrequent exacerbations (0.3% and 0.4%) (**Supplementary Table 4**). There was also an increase in the other asthma medications used from the baseline to follow-up periods when categorized by exacerbation frequency, with SABA use increasing in patients with frequent exacerbations from 61.9 to 86.3% and increasing in patients with infrequent exacerbations from 43.1 to 56.7%. Of note, OCS use increased from the baseline to follow-up period from 56.3 to 97.5% in patients with frequent exacerbations and from 25.4 to 33.1% in patients with infrequent exacerbations (**Supplementary Table 4**).

## Discussion

The measurement of blood eosinophil count, the most established predictive biomarker for targeted biologic treatment response [13], is an important step towards the phenotypic characterization of patients with asthma and assessing targeted biologic therapy eligibility [16]. This large, retrospective study provided insight into the real-world prevalence of blood eosinophil count testing for patients with asthma in the US. Results of this study show that regardless of exacerbation frequency or HCP type, approximately 70% of patients are likely to have blood eosinophil counts ≥ 150 cells/µL, making them potentially eligible for targeted biologic therapy. Furthermore, more than one-third of patients could be considered to be in a highly responsive subgroup for this therapy, with blood eosinophil counts ≥ 300 cells/µL [8, 17, 18]. Although Allergists or Pulmonologists are more likely to perform blood eosinophil count tests compared with other HCPs, particularly in patients with frequent exacerbations, only two-thirds of patients are being tested, which could limit access to targeted biologic therapy. Overall, these results highlight that despite patients having a high burden of disease, indicated by the common comorbidities, exacerbation frequencies and high OCS dependencies, blood eosinophil count tests are suboptimal.

Of the 400,254 patients included in this study, over three-quarters (77%) were cared for by a PCP, with 17% in the care of a specialist Allergist/Pulmonologist. Overall, demographics in this study population were consistent with previous asthma real-world studies [10, 16, 17], with a mean age of 51 years and a higher predominance of asthma in females (70.8%). While baseline demographics and clinical characteristics were generally similar between patients by exacerbation frequency, clear differences were seen when analyzed by HCP type. Patients with asthma under Allergist/Pulmonologist care were typically older and had more frequent exacerbations throughout the study compared with those under PCP or other HCP care. This is potentially a reflection of the time it takes for patients with severe asthma to be referred to a specialist for treatment and the severity of their disease once referred. Accordingly, patients under the care of an Allergist/Pulmonologist also had higher comorbidity index scores and more frequently had the top 10 comorbidities assessed in the study, with over half of patients in this subgroup having comorbid hyper-tension. Additionally, patients under the care of an Allergist/Pulmonologist had the greatest use of other asthma medications (ICS, OCS, SABA and SAMA) in the baseline and follow-up periods compared with patients under the care of a PCP or other HCP. Together, these results suggest that patients with a greater burden of asthma-specific and unrelated comorbidities may have asthma that is more severe and difficult to treat and are more likely to be referred for specialist care.

Analysis of demographics by HCP types also highlighted potential differences in access to healthcare among racial and ethnic groups. Observation of trends among ethnic subgroups highlighted that fewer African American and Hispanic/Latino patients received specialist Allergist/Pulmonologist care than Caucasian patients. This is consistent with previous reports of disparities in access to specialty asthma healthcare among African American and Hispanic/Latino communities and may result in the under-prescription of targeted asthma therapy to these patient subgroups, along with overall increased morbidity [19].

Although patients under Allergist/Pulmonologist care more frequently had blood eosinophil counts performed during the baseline or follow-up periods, compared with other HCPs, approximately one-third of all patients did not have the test performed. An unexpected finding in the current study was that within the subgroup populations, a slightly lower proportion of patients in the Allergist/Pulmonologist and PCP subgroups (64% and 63%, respectively) had a blood eosinophil count or CBC count test on follow-up, compared with patients in the other HCP provider subgroup (68%). The other HCP subgroup included 98 different specialties with a preponderance of hospital-based specialist services; however, it is unclear why this group had the lowest proportion of patients with blood eosinophil counts at baseline, but amongst the highest rates of testing at follow-up. Testing was even less frequent in patients who were under the care of a PCP: between 32% and 44% of patients did not have a blood eosinophil count test performed. Additionally, 3–45% and 25–35% of patients with frequent exacerbations did not have a blood eosinophil count ordered in the baseline and follow-up periods, respectively, across HCP subgroups. Consequently, up to approximately half of patients with frequent exacerbations who may be eligible for targeted biologic therapies are not being assessed for eligibility. It is important to note that the proportion of patients determined in this study to have had eosinophil counts measured may be an underestimation, due to the limitations of the analysis or measurement of eosinophilic inflammation by alternative methods such as with fractional exhaled nitric oxide levels. However, clinicians who use these alternative testing strategies may also check blood eosinophil counts.

Although associated with greater healthcare resource use for patients [20], blood eosinophil count testing and referral to specialist care settings are important to ensure that patients with severe asthma are not being undertreated or are over-reliant on OCS use [21]. Consequently, current GINA recommendations suggest that patients with asthma under specialist care undergo blood eosinophil count/CBC testing, among other parameters [1]. Overall, there was no difference in blood eosinophil count tests ordered between patients with frequent and infrequent exacerbations; in patients with frequent exacerbations, this finding was despite half of patients using OCS during baseline and nearly all patients using OCS during follow-up, highlighting the unmet needs of these patients. Moreover, for patients under the care of all HCP types, there was an increase in other asthma medication use including ICS, OCS, SABA and SAMA from baseline to follow-up. Implementing blood eosinophil count testing in primary care could prove useful in identifying a subset of patients who would benefit from referral to an asthma specialist and targeted treatment. This is particularly evident in the context of the large proportion of patients receiving care with a PCP (77%) or other HCP (7%) compared with specialist care (16%). Consequently, this represents a considerable population of patients who could benefit from earlier blood eosinophil count testing and subsequently specialist care and/or targeted treatments [1]. PCP care is important for patients with asthma to reduce rates of uncontrolled asthma due to poor adherence; this should be supported in tandem with seeking appropriate specialist care [22]. Future research should also consider analyzing eosinophil testing rates by patient outcomes.

Regardless of HCP type, more than half of patients had a maximum blood eosinophil count ≥ 150 cells/μL, with higher counts more commonly in patients who experienced frequent exacerbations. Specifically, approximately half of patients with frequent exacerbations in the follow-up period had maximum blood eosinophil count values ≥ 300 cells/µL. This is consistent with previous evidence suggesting patients with moderate-to-severe asthma are more likely to present with an eosinophilic phenotype compared with patients with mild asthma [23]. This suggests that a considerable proportion of patients with asthma are not receiving tests despite potentially being eligible for additional treatment options available to them [1]. Accordingly, even though a larger number of patients under Allergist/Pulmonologist care received targeted biologic therapy compared with other HCPs, the absolute proportions were low (~ 2%) during the baseline and follow-up periods. However, as this study did not require evidence of targeted biologic therapy use for patient selection, this may have resulted in the underestimation of the prevalence of targeted therapies, making causality difficult to establish. Although conclusions cannot be definitively drawn from this study regarding the levels of targeted biologic therapy use by HCP subgroups due to the study design, this finding may reflect that some biologic therapy treatments for severe (eosinophilic) asthma including mepolizumab [24], benralizumab [25], reslizumab [26], and dupilumab [27] were only approved during the study period. Omalizumab, however, was approved before the study period and was found to be the most frequently used biologic. Given serum IgE levels are tested to guide omalizumab dosing in patients with severe asthma and blood eosinophil count is not required for treatment, this may have resulted in a lower prevalence of blood eosinophil count testing than may be expected if this study was repeated following these more recent approvals. The biologic approvals were based on demonstrated efficacy in reducing exacerbation rates, reducing OCS use and improving HRQoL in both clinical trials and real-world settings [6–11, 14, 17, 28]. Greater efficacy (reduction of asthma exacerbation) of asthma biologics in patients with higher blood eosinophil counts has also been demonstrated in clinical studies [7, 8, 29–31].

Overall, these data support the recent study from Heaney et al., [4] which reported that a larger proportion of patients with severe asthma may have an eosinophilic phenotype than previously suggested. As per the GINA guidelines, blood eosinophil counts can vary over time and repeated testing measures are required to ensure accurate phenotyping of eosinophilic asthma [1]. The Heaney et al. study considered patients with asthma who had a maximum blood eosinophil count of ≥ 300 cells/μL or blood eosinophil count of ≥ 150–300 cells/μL with OCS use or a combination of comorbidities classified as eosinophilic; within this study, 36–59% of patients had a maximum blood eosinophil count ≥ 300 cells/μL, with a further 22–31% having a maximum blood eosinophil count ≥ 150–300 cells/µL [4]. In the present study, the findings were similar, with the proportion of patients who had a maximum blood eosinophil count of ≥ 300 cells/µL ranging from 36 to 53% across patients and HCP subtypes at baseline and from 38 to 59% across exacerbation frequency and HCP subtypes during the follow-up period.

There are several inherent limitations to this database-based study, which should be considered when interpreting the results. First, this study is limited to only those patients seeking care with providers covered by the Explorys^®^ Universe Database system. As a consequence, the results may not be generalizable to all patients with asthma, for example, most patients within the database were located in the North Central region of the US, so these results may not be representative of a more broadly distributed population of patients with asthma in the US. Additionally, if testing was performed outside of the integrated delivery networks which provide data to Explorys^®^, the results may not have been captured. Second, the study was claims-based in nature and may include coding limitations and/or data entry errors. Third, these data were calculated as aggregate results, therefore determining how many patients who were not blood eosinophil count/CBC tested during the baseline period then subsequently tested during the follow-up period was not possible. Fourth, this study period concluded in 2019, therefore these data may not capture the current prescribing levels of the more recently available targeted asthma therapies, and may not reflect GINA guideline changes including the requirement of patients to have blood eosinophil count tests. Furthermore, as mepolizumab, benralizumab, dupilumab, and reslizumab were newly approved at the time of the study period, the data captured here may not reflect the current levels of blood eosinophil count testing used by physicians to determine eligibility for these now well-established biologic therapies. Fifth, for patients who experienced comorbidities such as atopic dermatitis, COPD or rheumatoid arthritis, the use of CBCs with differential to assess and manage these conditions may make blood eosinophil counts more readily available. Similarly, since biologics were approved for other conditions such as atopic dermatitis during the study period, prescriptions for targeted biologic therapies may not be exclusively for severe asthma. Sixth, while availability of laboratory data and evidence of outpatient utilization were required for inclusion in the study, the availability of prescribed medication for inclusion in the study was not required. Therefore, asthma medication utilization is likely an underestimate. However, this underreporting is unlikely to differ by HCP type. Finally, blood eosinophil counts may have been assessed outside of the study period or through another method not captured by this study design; however, while analysis of blood eosinophil counts are a recent development, an eosinophilic phenotype (≥ 150 cells/µL) appears to be associated with increased healthcare usage, making it likely that a high proportion of these were captured in this study [20].

## Conclusions

The purpose of this retrospective study was to evaluate the real-world prevalence of blood eosinophil count testing undertaken for patients with asthma and compare this across different HCP types and exacerbation frequencies. Although a greater number of patients under the care of an Allergist or Pulmonologist had blood eosinophil testing completed compared with patients under the care of a PCP, based on the large proportion of patients with frequent exacerbations at follow-up remaining under the care of a PCP, a sizeable population of patients are likely being under-tested. Therefore, all HCPs should consider increasing routine blood eosinophil count tests for patients with asthma, which would enhance the identification of patients with an eosinophilic asthma phenotype. Given their increased accessibility to patients, PCPs in particular play an important role in facilitating the uptake of this testing. These measures could improve the likelihood of patients accessing phenotype-specific treatment.

## Electronic supplementary material

Below is the link to the electronic supplementary material.

**Supplementary Material 1: Supplementary Table 1.** Patient eligibility codes.

**Supplementary Material 2: Supplementary Table 2.** Laboratory codes.

**Supplementary Material 3: Supplementary Table 3.** Systemic corticosteroid drug names included within study.

**Supplementary Material 4: Supplementary Table 4.** Asthma medication use by exacerbation frequency.

**Supplementary Material 5: Supplementary Figure 1.** Study sample selection.

## Data Availability

To access data supporting this study and related study documents, please submit a request via https://www.gsk-studyregister.com/en/.

## Abbreviations

CBC: Complete blood count
COPD: Chronic obstructive pulmonary disease
CPT: Current procedural terminology
DCI: Deyo-Charlson Comorbidity Index
GINA: Global initiative for asthma
HCP: Healthcare provider
HRQoL: Health-related quality of life
ICD: International classification of disease
ICS: Inhaled corticosteroids
IM: Intramuscular
IV: Intravenous
LABA: Long-acting β2-agonist
LAMA: Long-acting muscarinic agonist
LOINC: Logical observation identifiers names and codes
LTRA: Leukotriene receptor antagonist
OCS: Oral corticosteroids
PCP: Primary care physician
SABA: Short-acting β2-agonist
SAMA: Short-acting muscarinic antagonist
SD: Standard deviation
SNOMED: Systematized Nomenclature of Medicine

## References

[1] GINA. Global strategy for asthma management and prevention. [Internet]. 2022 [cited 2023 Feb 1]: Available from: https://ginasthma.org/wp-content/uploads/2022/07/GINA-Main-Report-2022-FINAL-22-07-01-WMS.pdf

[2] Tran TN, Kerkhof M, Carter V, Price DB. Persistence of eosinophilic asthma endotype and clinical outcomes: a real-world observational study. J Asthma Allergy. 2021;14:727–42. 34211281 10.2147/JAA.S306416 PMC8242130

[3] Tupper OD, Ulrik CS. Long-term predictors of severe exacerbations and mortality in a cohort of well-characterised adults with asthma. Respir Res. 2021;22(1):269. 34670588 10.1186/s12931-021-01864-z PMC8529759

[4] Heaney LG, Perez de Llano L, Al-Ahmad M, Backer V, Busby J, Canonica GW, et al. Eosinophilic and noneosinophilic asthma: an Expert Consensus Framework to characterize phenotypes in a global real-life severe asthma cohort. Chest. 2021;160(3):814–30. 33887242 10.1016/j.chest.2021.04.013

[5] Walford HH, Doherty TA. Diagnosis and management of eosinophilic asthma: a US perspective. J Asthma Allergy. 2014;7:53–65. 24748808 10.2147/JAA.S39119 PMC3990389

[6] Bel EH, Wenzel SE, Thompson PJ, Prazma CM, Keene ON, Yancey SW, et al. Oral glucocorticoid-sparing effect of mepolizumab in eosinophilic asthma. N Engl J Med. 2014;371(13):1189–97. 25199060 10.1056/NEJMoa1403291

[7] Castro M, Zangrilli J, Wechsler ME, Bateman ED, Brusselle GG, Bardin P, et al. Reslizumab for inadequately controlled asthma with elevated blood eosinophil counts: results from two multicentre, parallel, double-blind, randomised, placebo-controlled, phase 3 trials. Lancet Respir Med. 2015;3(5):355–66. 25736990 10.1016/S2213-2600(15)00042-9

[8] FitzGerald JM, Bleecker ER, Menzies-Gow A, Zangrilli JG, Hirsch I, Metcalfe P, et al. Predictors of enhanced response with benralizumab for patients with severe asthma: pooled analysis of the SIROCCO and CALIMA studies. Lancet Respir Med. 2018;6(1):51–64. 28919200 10.1016/S2213-2600(17)30344-2

[9] FitzGerald JM, Bleecker ER, Nair P, Korn S, Ohta K, Lommatzsch M, et al. Benralizumab, an anti-interleukin-5 receptor alpha monoclonal antibody, as add-on treatment for patients with severe, uncontrolled, eosinophilic asthma (CALIMA): a randomised, double-blind, placebo-controlled phase 3 trial. Lancet. 2016;388(10056):2128–41. 27609406 10.1016/S0140-6736(16)31322-8

[10] Ortega HG, Liu MC, Pavord ID, Brusselle GG, FitzGerald JM, Chetta A, et al. Mepolizumab treatment in patients with severe eosinophilic asthma. N Engl J Med. 2014;371(13):1198–207. 25199059 10.1056/NEJMoa1403290

[11] Pavord ID, Korn S, Howarth P, Bleecker ER, Buhl R, Keene ON, et al. Mepolizumab for severe eosinophilic asthma (DREAM): a multicentre, double-blind, placebo-controlled trial. Lancet. 2012;380(9842):651–9. 22901886 10.1016/S0140-6736(12)60988-X

[12] Rogliani P, Calzetta L, Matera MG, Laitano R, Ritondo BL, Hanania NA, et al. Severe asthma and biological therapy: when, which, and for whom. Pulm Ther. 2020;6(1):47–66. 32048241 10.1007/s41030-019-00109-1 PMC7229123

[13] Runnstrom M, Pitner H, Xu J, Lee FE, Kuruvilla M. Utilizing predictive inflammatory markers for guiding the use of biologicals in severe asthma. J Inflamm Res. 2022;15:241–9. 35068937 10.2147/JIR.S269297 PMC8769207

[14] Chupp GL, Bradford ES, Albers FC, Bratton DJ, Wang-Jairaj J, Nelsen LM, et al. Efficacy of mepolizumab add-on therapy on health-related quality of life and markers of asthma control in severe eosinophilic asthma (MUSCA): a randomised, double-blind, placebo-controlled, parallel-group, multicentre, phase 3b trial. Lancet Respir Med. 2017;5(5):390–400. 28395936 10.1016/S2213-2600(17)30125-X

[15] Reddel HK, Taylor DR, Bateman ED, Boulet LP, Boushey HA, Busse WW, et al. An official American Thoracic Society/European Respiratory Society statement: asthma control and exacerbations: standardizing endpoints for clinical asthma trials and clinical practice. Am J Respir Crit Care Med. 2009;180(1):59–99. 19535666 10.1164/rccm.200801-060ST

[16] Azim A, Newell C, Barber C, Harvey M, Knight D, Freeman A, et al. Clinical evaluation of type 2 disease status in a real-world population of difficult to manage asthma using historic electronic healthcare records of blood eosinophil counts. Clin Experimental Allergy. 2021;51(6):811–20. 10.1111/cea.13841 33528864

[17] Harrison T, Canonica GW, Chupp G, Lee J, Schleich F, Welte T et al. Real-world mepolizumab in the prospective severe asthma REALITI-A study: initial analysis. Eur Respir J. 2020;56(4). 10.1183/13993003.00151-2020 PMC7559868 32817259

[18] Albers FC, Licskai C, Chanez P, Bratton DJ, Bradford ES, Yancey SW, et al. Baseline blood eosinophil count as a predictor of treatment response to the licensed dose of mepolizumab in severe eosinophilic asthma. Respir Med. 2019;159:105806. 31751853 10.1016/j.rmed.2019.105806

[19] Grant T, Croce E, Matsui EC. Asthma and the social determinants of health. Ann Allergy Asthma Immunol. 2022;128(1):5–11. 34673220 10.1016/j.anai.2021.10.002 PMC8671352

[20] Dotiwala Z, Casciano J, Davis JR, Fox K, Gopalan G, Rastogi S, et al. Effect of clinically significant thresholds of eosinophil elevation on health care resource use in asthma. Ann Allergy Asthma Immunol. 2020;125(2):182–9. 32371242 10.1016/j.anai.2020.04.024

[21] Graff S, Vanwynsberghe S, Brusselle G, Hanon S, Sohy C, Dupont LJ, et al. Chronic oral corticosteroids use and persistent eosinophilia in severe asthmatics from the Belgian severe asthma registry. Respir Res. 2020;21(1):214. 32787967 10.1186/s12931-020-01460-7 PMC7424982

[22] Yawn BP. The role of the primary care physician in helping adolescent and adult patients improve asthma control. Mayo Clin Proc. 2011;86(9):894–902. 21878602 10.4065/mcp.2011.0035 PMC3257999

[23] Casciano J, Krishnan JA, Small MB, Buck PO, Gopalan G, Li C, et al. Value of peripheral blood eosinophil markers to predict severity of asthma. BMC Pulm Med. 2016;16(1):109. 27473851 10.1186/s12890-016-0271-8 PMC4966857

[24] GSK. NUCALA Prescribing Information (US). 2022 [updated 2022 Jan; cited 2022 July 18]. Available from: https://gskpro.com/content/dam/global/hcpportal/en_US/Prescribing_Information/Nucala/pdf/NUCALA-PI-PIL-IFU-COMBINED.PDF

[25] Astrazeneca. FASENRA Prescribing Information [Internet]. 2022 [updated 2021 Feb; cited 2022 Nov 9]. https://den8dhaj6zs0e.cloudfront.net/50fd68b9-106b-4550-b5d0-12b045f8b184/3647bed4-ce91-4fe7-9bc5-32dbee73f80a/3647bed4-ce91-4fe7-9bc5-32dbee73f80a_viewable_rendition__v.pdf

[26] Teva Pharmaceutical Industries Ltd. CINQAIR^®^ (reslizumab) Prescribing Information 2016. https://www.accessdata.fda.gov/drugsatfda_docs/label/2016/761033lbl.pdf

[27] Sanofi Ra. DUPIXENT Prescribing information (US) [Internet]. 2022 [updated 2022 Oct; cited 2023 Feb 1]. Available from: https://www.regeneron.com/downloads/dupixent_fpi.pdf

[28] Bousquet J, Humbert M, Gibson PG, Kostikas K, Jaumont X, Pfister P, et al. Real-world effectiveness of omalizumab in severe allergic asthma: a meta-analysis of observational studies. J Allergy Clin Immunol Pract. 2021;9(7):2702–14. 33486142 10.1016/j.jaip.2021.01.011

[29] Ortega HG, Yancey SW, Mayer B, Gunsoy NB, Keene ON, Bleecker ER, et al. Severe eosinophilic asthma treated with mepolizumab stratified by baseline eosinophil thresholds: a secondary analysis of the DREAM and MENSA studies. Lancet Respir Med. 2016;4(7):549–56. 27177493 10.1016/S2213-2600(16)30031-5

[30] Busse W, Spector S, Rosen K, Wang Y, Alpan O. High eosinophil count: a potential biomarker for assessing successful omalizumab treatment effects. J Allergy Clin Immunol. 2013;132(2):485–e611. 23591271 10.1016/j.jaci.2013.02.032

[31] Castro M, Corren J, Pavord ID, Maspero J, Wenzel S, Rabe KF, et al. Dupilumab Efficacy and Safety in Moderate-to-severe uncontrolled asthma. N Engl J Med. 2018;378(26):2486–96. 29782217 10.1056/NEJMoa1804092

