# Supplementary material for: Eosinophil count testing in patients with asthma varies by healthcare provider type in the US: a retrospective study
Source: Allergy Asthma Clin Immunol. 2024 Oct 24;20:56. doi: 10.1186/s13223-024-00917-4 (PMC11515424; doi:10.1186/s13223-024-00917-4)
Supplement: Supplementary file 1 — Supplementary Material 1: Supplementary Table 1. Patient eligibility codes. [file 13223_2024_917_MOESM1_ESM.docx]

**Table S1** Patient eligibility codes

| **Condition** | **Code type** | **Code** |
| --- | --- | --- |
| Extrinsic asthma | ICD-9-CM DX  SNOMED | 49300–302, 63088003, 91340006, 233681001, 233683003, 233688007, 389145006, 424643009, 703953004, 703954005, 708093000, 708095007, 735587000, 735588005, 762521001, 782513000, 782520007, 829976001 |
| Intrinsic asthma | ICD-9-CM DX  SNOMED | 49310–312, 12428000, 59327009, 92807009, 93432008, 233685005, 233686006, 266361008, 407674008, 423889005, 424199006, 445427006, 708094006, 708096008, 735589002, |
| Chronic obstructive asthma | ICD-9-CM DX  SNOMED | 49320–322, 1751000119100, 1761000119103, 10692721000119102 |
| Exercise induced bronchospasm | ICD-9-CM DX  ICD-10-CM DX  SNOMED | 49381, J45990 31387002 |
| Cough variant asthma | ICD-9-CM DX  ICD-10-CM DX  SNOMED | 49382, J45991, 409663006 |
| Asthma, unspecified type | ICD-9-CM DX | 49390–392 |
| Mild intermittent asthma | ICD-10-CM DX  SNOMED | J4520–522, 427603009, 427679007, 641000119106, 1741000119102, 125021000119107, 10674991000119104 |
| Mild persistent asthma | ICD-10-CM DX  SNOMED | J4530–532, 370218001, 426979002, 707445000, 7075110009, 707981009, 125011000119100, 135181000119109, 10675871000119106, 10675911000119109, 10675991000119100, 10676031000119106, 10676071000119109, 10676111000119102, 10676151000119101, 10676191000119106, 10676231000119102 |
| Moderate persistent asthma | ICD-10-CM DX  SNOMED | J4540–542, 370219009, 427295004, 707446004, 707512002, 707980005, 125001000119103, 135171000119106, 10676351000119103, 10676391000119108, 10676431000119103, 10676511000119109, 10676551000119105, 10676591000119100, 10676631000119100, 10676671000119102, 10676711000119103 |
| Severe persistent asthma | ICD-10-CM DX  SNOMED | J4550–552, 57546000, 370221004, 426656000, 707447008, 707513007, 707979007, 733858005, 124991000119109, 10675391000119101, 10675431000119106, 10675471000119109, 10675551000119104, 10675591000119109, 10675631000119109, 10675671000119107, 10675711000119106, 10675751000119107 |
| Unspecified asthma | ICD-10-CM DX  SNOMED | J45901, J45902, J45909, 30352005, 55570000, , 170631002, 170632009, 170633004, 170634005, 170635006, 170636007, 170637003, 170638008, 170655007, 170656008, 170657004, 170658009, 185728001, 185730004, 195967001, 195977004, 266364000, 270442000, 281239006, 304527002, 308501009, 312453004, 370202007, 370203002, 370204008, 370205009, 370206005, 370207001, 370208006, 370220003, 370225008, 370226009, 373899003, 390921001, 390940007, 395022009, 401193004, 425969006, 427354000, 473391009, 707444001, 708038006, 708090002, 733858005, 734904007, 734905008, 782559003, 786836003, 401000119107, 5281000124103, 99031000119107, 2360001000004109, 10674711000119105, |
| Occupational asthma | SNOMED | 11641008, 18041002, 19849005, 34015007, 56968009, 57607007, 233687002, 404804003, 404806001, 404808000, 418395004 |
| Other asthma | ICD-10-CM DX  SNOMED | J45998, 195949008, 225057002, 233678006, 233679003, 405944004, 442025000, 72301000119103, 10742121000119104, 16055311000119107, 16584951000119101 |
| Other interstitial pulmonary disease | SNOMED | 41997000 |

COPD, chronic obstructive pulmonary disease; ICD-9-CM, international classification of diseases, ninth revision, clinical modification; ICD-10-CM, international classification of diseases, tenth revision, clinical modification.
