# Supplementary material for: Eosinophil count testing in patients with asthma varies by healthcare provider type in the US: a retrospective study
Source: Allergy Asthma Clin Immunol. 2024 Oct 24;20:56. doi: 10.1186/s13223-024-00917-4 (PMC11515424; doi:10.1186/s13223-024-00917-4)
Supplement: Supplementary file 2 — Supplementary Material 2: Supplementary Table 2. Laboratory codes. [file 13223_2024_917_MOESM2_ESM.docx]

### Table S2 Laboratory codes

| **Test** | **Code type** | **Code** |
| --- | --- | --- |
| CBC | LOINC  SNOMED  CPT | 57021-8, 57022-6, 57782-5, 58410-2, 69742-5, 9564003, 35774004, 85048, 85004 |
| WBC | LOINC | 26464-8, 6690-2, 804-5, 729-4 |
| BEC | LOINC  SNOMED | 32349-3, 32154-7, 32350-1, 32155-4, 26449-9, 711-2, 712-0, 26450-7, 713-8, 714-6, 71960002, 142932005, 142936008, 143123003, 165525005, 165719008, 310540006 |

BEC, blood eosinophil count; CBC, complete blood count; CPT, current procedural terminology; LOINC, logical observation identifier names and codes; SNOMED, systematized nomenclature of medicine clinical terms; WBC, white blood cell count.
