# Supplementary material for: Eosinophil count testing in patients with asthma varies by healthcare provider type in the US: a retrospective study
Source: Allergy Asthma Clin Immunol. 2024 Oct 24;20:56. doi: 10.1186/s13223-024-00917-4 (PMC11515424; doi:10.1186/s13223-024-00917-4)
Supplement: Supplementary file 3 — Supplementary Material 3: Supplementary Table 3. Systemic corticosteroid drug names included within study. [file 13223_2024_917_MOESM3_ESM.docx]

**Table S3** Systemic corticosteroid generic drug names included within study

| Betamethasone |
| --- |
| Betamethasone Ace/Betamethasone Na Phos |
| Betamethasone Sodium Phosphate |
| Budesonide |
| Budesonide, Micronized |
| Cortisone Acetate |
| Dexamethasone |
| Dexamethasone Acetate |
| Dexamethasone Sodium Phosphate |
| Dexamethasone Sodium Phosphate/Dextrose |
| Dexamethasone Sodium Phosphate/Sodium Chloride |
| Fludrocortisone Acetate |
| Hydrocortisone |
| Hydrocortisone Acetate |
| Hydrocortisone Cypionate |
| Hydrocortisone Sodium Phosphate |
| Hydrocortisone Sodium Succinate |
| Methylprednisolone |
| Methylprednisolone Acetate |
| Methylprednisolone Sodium Succinate |
| Prednis Acet.-Prednis Sod Phos |
| Prednisolone |
| Prednisolone Acetate |
| Prednisolone Acetate/Prednisolone Sodium Phosphate |
| Prednisolone Sodium Phosphate |
| Prednisolone Sodium Succinate |
| Prednisolone Tebutate |
| Prednisone |
| Triamcinolone |
| Triamcinolone Acetonide |
| Triamcinolone Diacetate |
| Triamcinolone Hexacetonide |
