# Supplementary material for: Eosinophil count testing in patients with asthma varies by healthcare provider type in the US: a retrospective study
Source: Allergy Asthma Clin Immunol. 2024 Oct 24;20:56. doi: 10.1186/s13223-024-00917-4 (PMC11515424; doi:10.1186/s13223-024-00917-4)
Supplement: Supplementary file 4 — Supplementary Material 4: Supplementary Table 4. Asthma medication use by exacerbation frequency. [file 13223_2024_917_MOESM4_ESM.docx]

**Table S4** Asthma medication use by exacerbation frequency

|  | **Total population**  **(n=400,254)** | | **Patients with infrequent (<2) exacerbations***  **(n=374,657)** | | **Patients with frequent (≥2) exacerbations***  **(n=25,597)** | |
| --- | --- | --- | --- | --- | --- | --- |
|  | **Baseline period** | **Follow-up period** | **Baseline period** | **Follow-up period** | **Baseline period** | **Follow-up period** |
| **Asthma-related biologics, n, (%)** | 1507 (0.4) | 2252 (0.6) | 1191 (0.3) | 1687 (0.5) | 316 (1.2) | 565 (2.2) |
| Benralizumab | 1 (0.0) | 40 (0.0) | 1 (0.0) | 32 (0.0) | 0 (0.0) | 8 (0.0) |
| Dupilumab | 4 (0.0) | 42 (0.0) | 4 (0.0) | 35 (0.0) | 0 (0.0) | 7 (0.0) |
| Mepolizumab | 29 (0.0) | 362 (0.1) | 27 (0.0) | 236 (0.1) | 2 (0.0) | 126 (0.5) |
| Omalizumab | 1473 (0.4) | 1847 (0.5) | 1159 (0.3) | 1403 (0.4) | 314 (1.2) | 444 (1.7) |
| Reslizumab | 1 (0.0) | 12 (0.0) | 1 (0.0) | 9 (0.0) | 0 (0.0) | 3 (0.0) |
| **Other asthma medications, n (%)** |  |  |  |  |  |  |
| ICS | 32,150 (8.0) | 47,092 (11.8) | 28,905 (7.7) | 41,060 (11.0) | 3245 (12.7) | 6032 (23.6) |
| OCS | 109,683 (27.4) | 149,162 (37.3) | 95,262 (25.4) | 124,195 (33.1) | 14,421 (56.3) | 24,967 (97.5) |
| SABA | 177,229 (44.3) | 234,642 (58.6) | 161,388 (43.1) | 212,561 (56.7) | 15,841 (61.9) | 22,081 (86.3) |
| SAMA | 12,435 (3.1) | 16,849 (4.2) | 10,399 (2.8) | 13,145 (3.5) | 2036 (8.0) | 3704 (14.5) |
| SABA + SAMA | 74,348 (18.6) | 103,691 (25.9) | 65,896 (17.6) | 89,467 (23.9) | 8452 (33.0) | 14,224 (55.6) |
| LABA | 2009 (0.5) | 2690 (0.7) | 1733 (0.5) | 2199 (0.6) | 276 (1.1) | 491 (1.9) |
| LAMA | 8585 (2.1) | 10,192 (2.5) | 7463 (2.0) | 8606 (2.3) | 1122 (4.4) | 1586 (6.2) |
| LTRA | 58,342 (14.6) | 80,737 (20.2) | 52,021 (13.9) | 70,594 (18.8) | 6321 (24.7) | 10,143 (39.6) |
| ICS/LABA | 60,130 (15.0) | 85,235 (21.3) | 55,513 (14.3) | 74,243 (19.8) | 6617 (25.9) | 10,992 (42.9) |
| Triple therapy (ICS+LABA+LAMA) | 39,483 (9.9) | 55,601 (13.9) | 35,370 (9.4) | 48,472 (12.9) | 4113 (16.1) | 7129 (27.9) |
| Mast cell stabilizers | 147 (0.0) | 194 (0.0) | 121 (0.0) | 152 (0.0) | 26 (0.1) | 42 (0.2) |
| Methylxanthines | 2,318 (0.6) | 2375 (0.6) | 1884 (0.5) | 1846 (0.5) | 434 (1.7) | 529 (2.1) |
| LABA/LAMA | 694 (0.2) | 1451 (0.4) | 638 (0.2) | 1279 (0.3) | 56 (0.2) | 172 (0.7) * |

*No statistical analysis was performed.

ICS, inhaled corticosteroids; LABA, long-acting beta agonist; LAMA, long-acting muscarinic antagonist; LTRA, leukotriene receptor antagonist; OCS, oral corticosteroids; SABA, Short-acting beta agonists; SAMA, short-acting muscarinic antagonist.
